# BRD4 inhibition alleviates mechanical stress-induced TMJ OA-like pathological changes and attenuates TREM1-mediated inflammatory response

**DOI:** 10.1186/s13148-021-01008-6

**Published:** 2021-01-15

**Authors:** Ziwei Huang, Ren Yang, Lu Zhang, Mengjiao Zhu, Caixia Zhang, Juan Wen, Huang Li

**Affiliations:** 1 Department of Orthodontics, Nanjing Stomatological Hospital, Medical School of Nanjing University, Nanjing, China; 2 Department of Orthodontics, School and Hospital of Stomatology, Tongji University, Shanghai Engineering Research Center of Tooth Restoration and Regeneration, Shanghai, China; 3 Department of Orthodontics, Nanjing Stomatological Hospital, Medical School of Nanjing University, 30 Central Road, Nanjing, 210008 China

**Keywords:** TMJ, Osteoarthritis, BRD4, TREM1, Inflammation

## Abstract

The aim of this paper was to investigate the protective effects of bromodomain containing 4 (BRD4) inhibition on the temporomandibular joint osteoarthritis (TMJ OA) induced by compressive mechanical stress and to explore the underlying mechanism. In vivo, a rat model of TMJ compressive loading device was used and BRD4 inhibitor was injected into the TMJ region. HE staining and micro-CT analysis were used for histological and radiographic assessment. Immunohistochemistry and qPCR were performed to detect inflammatory cytokines expressions. High-throughput ChIP-sequencing screening was performed to compare the BRD4 and H3K27ac binding patterns between condylar cartilage from control and mechanical force groups. In vitro, the mandibular condylar chondrocytes were treated with IL-1β. Small Interference RNA (siRNA) infection was used to silencing BRD4 or TREM1. qPCR was performed to detect inflammatory cytokines expressions. Our study showed that BRD4 inhibition can alleviate the thinning of condylar cartilage and subchondral bone resorption, as well as decrease the inflammatory factors expression both in vivo and in vitro. ChIP-seq analysis showed that BRD4 was more enriched in the promoter region of genes related to the stress and inflammatory pathways under mechanical stress in vivo. *Trem1*, a pro-inflammatory gene, was screened out from the overlapped BRD4 and H3K27ac increased binding sites, and *Trem1* mRNA was found to be regulated by BRD4 inhibition both in vivo and in vitro. TREM1 inhibition reduced the expression of inflammatory factors induced by IL-1β in vitro. In summary, we concluded that BRD4 inhibition can protect TMJ OA-like pathological changes induced by mechanical stress and attenuate TREM1-mediated inflammatory response.

## Introduction

Temporomandibular joint osteoarthritis (TMJ OA) is a traditionally defined degenerative joint disorder. It has been proved that excessive mechanical stress is an important etiological factor of TMJ OA [1–4]. We have successfully constructed the TMJ OA rat model induced by compressive mechanical stress [5–10]. Previous studies reported that the thinning of the cartilage, subchondral bone resorption, and extracellular matrix (ECM) damage are the main pathological changes induced by excessive mechanical stress [7–9]. Recently, researchers began to focus on the role of inflammation in TMJ OA [11–13]. From the traditionally defined degenerative disease to the present aseptic inflammatory disease, the mechanism of these pathological changes is still unclear.

More and more studies found that many human diseases are associated with epigenetic changes [13–15]. BRD4 is a double bromodomain protein of the BET family which binds the acetylated histones and regulates transcription as an epigenetic reader [16]. BRD4 is considered as a potential therapeutic target in many inflammatory diseases [17–22]. It was reported that BRD4 has dual effects on the HMGB1 and NF-κB signaling pathways [18] and BRD4 can modulate macrophage inflammatory responses [17]. BRD4 can also regulate osteoclast differentiation and was involved in the osteoporosis [14, 23, 24]. In recent years, several BRD4 inhibitors such as JQ1 were developed and proved to inhibit inflammation through blocking the interaction between BRD4 and acetylated histones [17–19, 25, 26]. Thereby, whether BRD4-mediated inflammatory response also plays a key role in TMJ OA sparked our interest.

Based on previous studies, we hypothesized that BRD4 inhibition may alleviate the TMJ OA-like pathological changes induced by excessive mechanical stress. To prove this hypothesis and explore the underlying mechanism, we designed in vitro IL-1β treatment model and in vivo rat model of TMJ compressive loading device with the use of BRD4 inhibitor JQ1 and siRNA interference. We found that BRD4 inhibition can alleviate the thinning of condylar cartilage expression and decrease the inflammatory factors. ChIP-seq analysis also showed that BRD4 was more enriched in the promoter region of genes related to the stress and inflammatory pathways under mechanical stress. *Trem1* was screened out from the overlapped binding sites of BRD4 and H3K27ac ChIP-seq. We demonstrated that BRD4 inhibition can reduce the TREM1-mediated inflammatory response after overloading mechanical stress or IL-1β treatment.

## Result

### BRD4 inhibition can relieve the condylar cartilage thinning and subchondral bone resorption induced by excessive mechanical stress in vivo

**Fig. 1:**
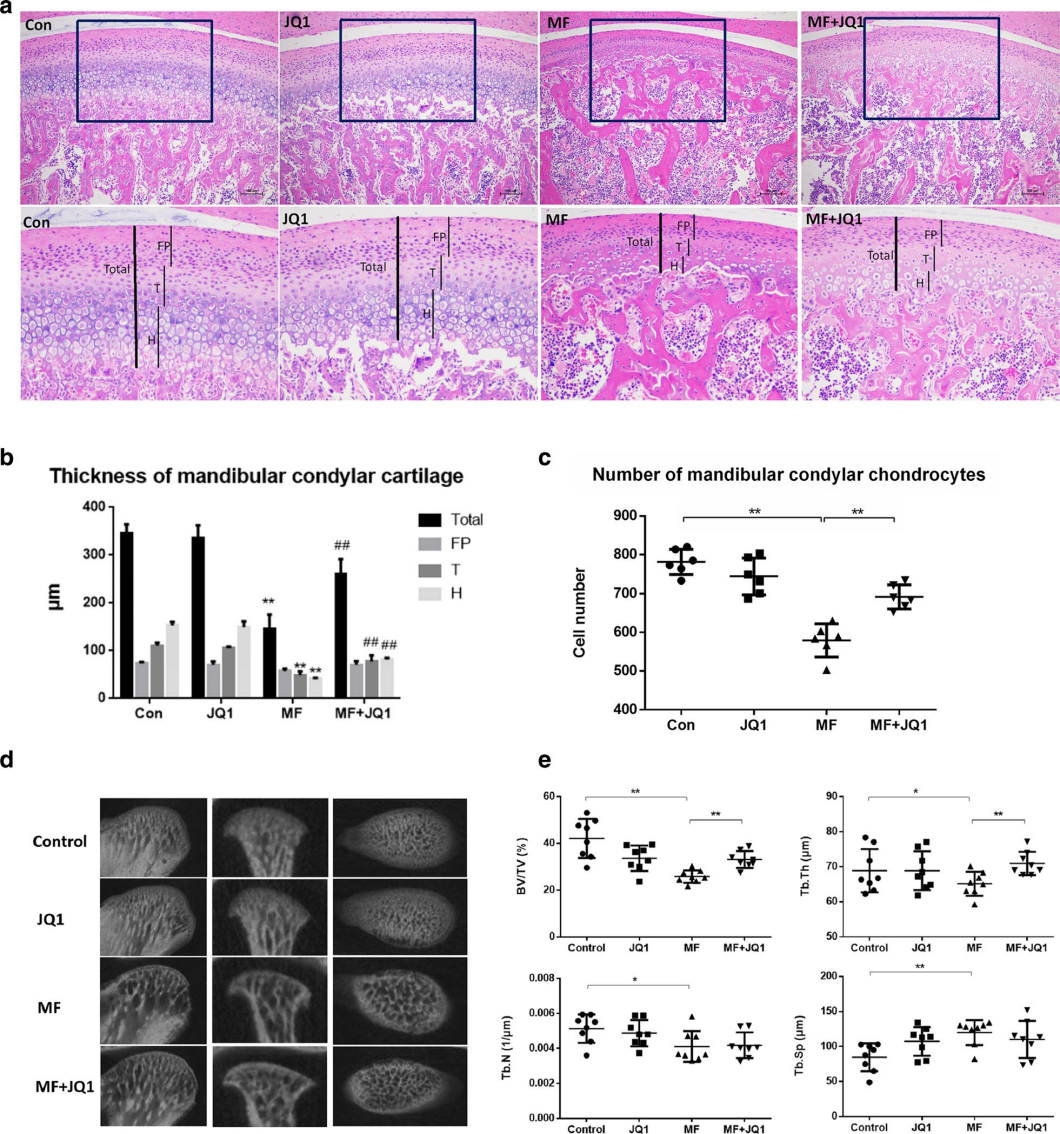
JQ1 can relieve the condylar cartilage thinning and subchondral bone resorption induced by overloading mechanical stress. **a** HE-stained sections (100×) of cartilage treated with 50 μM JQ1 at 14 days (*n* = 6). Scale bar indicates 100 μm. Cartilage thickness is indicated (black bar). FP indicates proliferative zone, T indicates transition zone, and H indicates hypertrophic zone. **b** Quantification of cartilage thickness of the samples shown in 1A (*n* = 6). Error bars indicate the standard deviation. (^##^*P* < 0.01 vs MF group; **P < 0.01 vs control group). **c** Quantification of chondrocytes of the samples shown in 1A (*n* = 6). Error bars indicate the standard deviation (*P < 0.05; **P < 0.01). **d** Representative images showing trabecular architecture by micro-computed tomography (mCT) reconstruction in the subchondral bone. **e** mCT measurements for the indicated parameters in the subchondral bone. BV/TV, Tb.Th, Tb.N, and Tb.Sp were determined by mCT analysis (*n* = 8). Error bars indicate the standard deviation. (*P < 0.05; **P < 0.01). MF, mechanical force

To explore whether BRD4 inhibition has a protective effect on cartilage under excessive mechanical stress, we set up 4 experimental groups including control group (Control), JQ1 group, mechanical force group (MF) and mechanical force plus JQ1 injection group (MF + JQ1) (Fig. 1a). JQ1 can block the interaction between BRD4 and acetylated histones and was used as the BRD4 inhibitor [27]. HE results showed that the control group had thicker cartilage and clear structure. The fibrous proliferative layer (FP), the transition layer (T), and the hypertrophic layer (H) were clearly layered, and the trabecular bone was dense in subchondral bone. The thickness and morphology of the cartilage in the JQ1 group were basically the same as those in the control group. After 14 days of overloading, the cartilage thickness reduced by 60% compared with the control group and apparent subchondral bone destruction was found with marked inflammatory cells infiltration near the dilated blood vessels (Fig. 1b). BRD4 inhibitor JQ1 can relieve the cartilage thinning induced by excessive mechanical stress. Consistent with the thickness of the cartilage, the number of chondrocytes also decreased in MF group and increased in MF + JQ1 groups compared to MF group (Fig. 1c). These results suggested that BRD4 inhibitor JQ1 can effectively reduce the condylar cartilage thinning and chondrocytes loss caused by excessive mechanical stress.

Micro-CT examination can reflect the changes of condylar subchondral bone morphology in a more stereoscopic and intuitive way from three dimensions (Fig. 1d). The results showed that the ratio of bone volume to tissue volume (BV/TV), trabecular number (Tb.N.), and trabecular thickness (Tb.Th.) decreased and trabecular separation (Tb.Sp.) increased in the MF group. In the MF + JQ1 group, the BV/TV increased by 27% (*P* < 0.01) and the Tb.Th increased by 9% compared with the MF group (Fig. 1e). It is indicated that JQ1 can inhibit the condylar subchondral bone resorption and is beneficial to maintain the normal morphological structure of subchondral bone under excessive mechanical stress.

### BRD4 inhibition can alleviate the expression of inflammatory factors induced by excessive mechanical stress in vivo and IL-1β in vitro

**Fig. 2:**
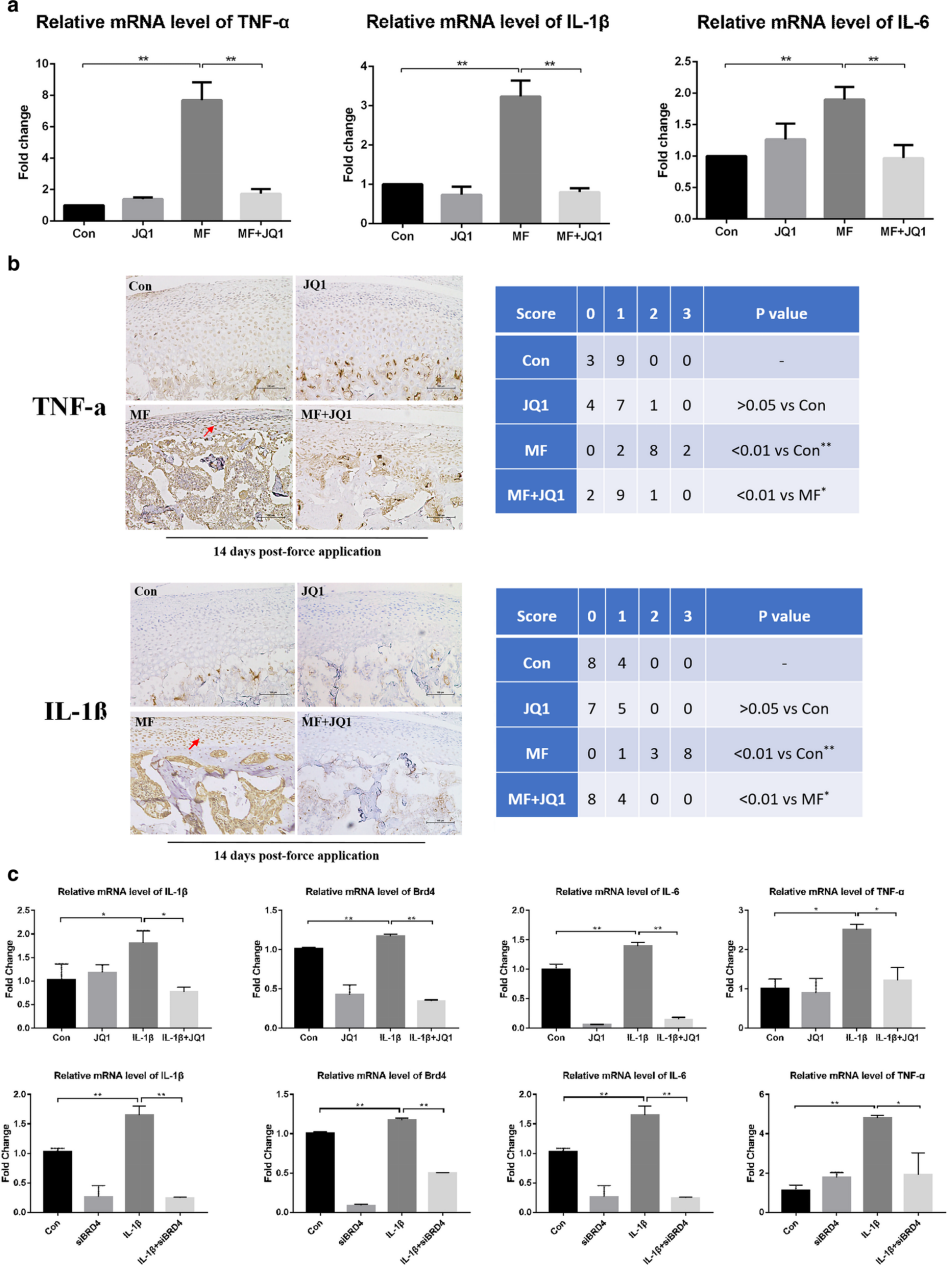
BRD4 inhibition can alleviate the expression of inflammatory factors induced by excessive mechanical stress and IL-1β. The concentration of JQ1 used in animal experiment is 50 μM. **a** qRT-PCR analysis of *Tnf-α, Il-1β*, and *Il-6* in condylar cartilage at 14 days (*n* = 6). **b** Immunohistochemical analysis of TNF-α and IL-1β in condylar cartilage (200×) at 14 days (*n* = 12). TNF-α expressing and IL-1β expressing chondrocytes are denoted (arrow). **c** qRT-PCR analysis of *Il-1β, Brd4, Il-6, Tnf-α* in chondrocytes from samples treated as indicated. Error bars indicate the standard deviation (*P < 0.05; **P < 0.01)

To confirm whether BRD4 inhibition can inhibit the cartilage inflammation induced by mechanical stress, we detected the expression of inflammatory factors by qRT-PCR and immunohistochemistry in vivo. The mRNA level of *Tnf-α, Il-1β* and *Il-6* showed no significant difference in the control group and JQ1 group. After 14 days of overloading, the above genes expression increased significantly. JQ1 can reduce the *Tnf-α, Il-1β*, and *Il-6* mRNA expression (Fig. 2a). Next, we performed TNF-α and IL-1β immunohistochemical staining and scoring. The results corresponded to those of qRT-PCR (Fig. 2b). These results indicated that BRD4 inhibitor JQ1 can inhibit the expression of inflammatory factors in rat condylar cartilage induced by mechanical stress. Previous studies have reported that IL-1β triggers pro-inflammatory factor production in chondrocytes [11, 28, 29] and IL-1β plays a vital role in TMJ OA development [11, 30]. Therefore, we used IL-1β as the stimuli to study the anti-inflammation effect of BRD4 inhibition in vitro. The efficiency of siBRD4 was detected as shown in Additional file 1: Fig. S2. We found that both JQ1 and siBRD4 can inhibit the increase of *Brd4, Il-6, Tnf-α* mRNA and endogenous *Il-1β* mRNA induced by IL-1β in vitro (Fig. 2c).

### Excessive mechanical stress increases BRD4 occupancy on inflammation-related genes promoter regions in vivo

Previously we demonstrated that BRD4 inhibition showed significant protective effect on cartilage thinning, subchondral bone resorption, and inflammatory factors expression induced by mechanical stress. As the mechanism of BRD4 inhibition is blocking the interaction between BRD4 and acetylated histones sites on DNA, we next attempt to investigate the BRD4 genome-wide occupancy profile to fully uncover the changes of BRD4′s binding to DNA under excessive mechanical stress. We performed chromatin immunoprecipitation sequencing (ChIP-seq) with BRD4 antibody using cartilage samples from control and MF groups.

**Fig. 3:**
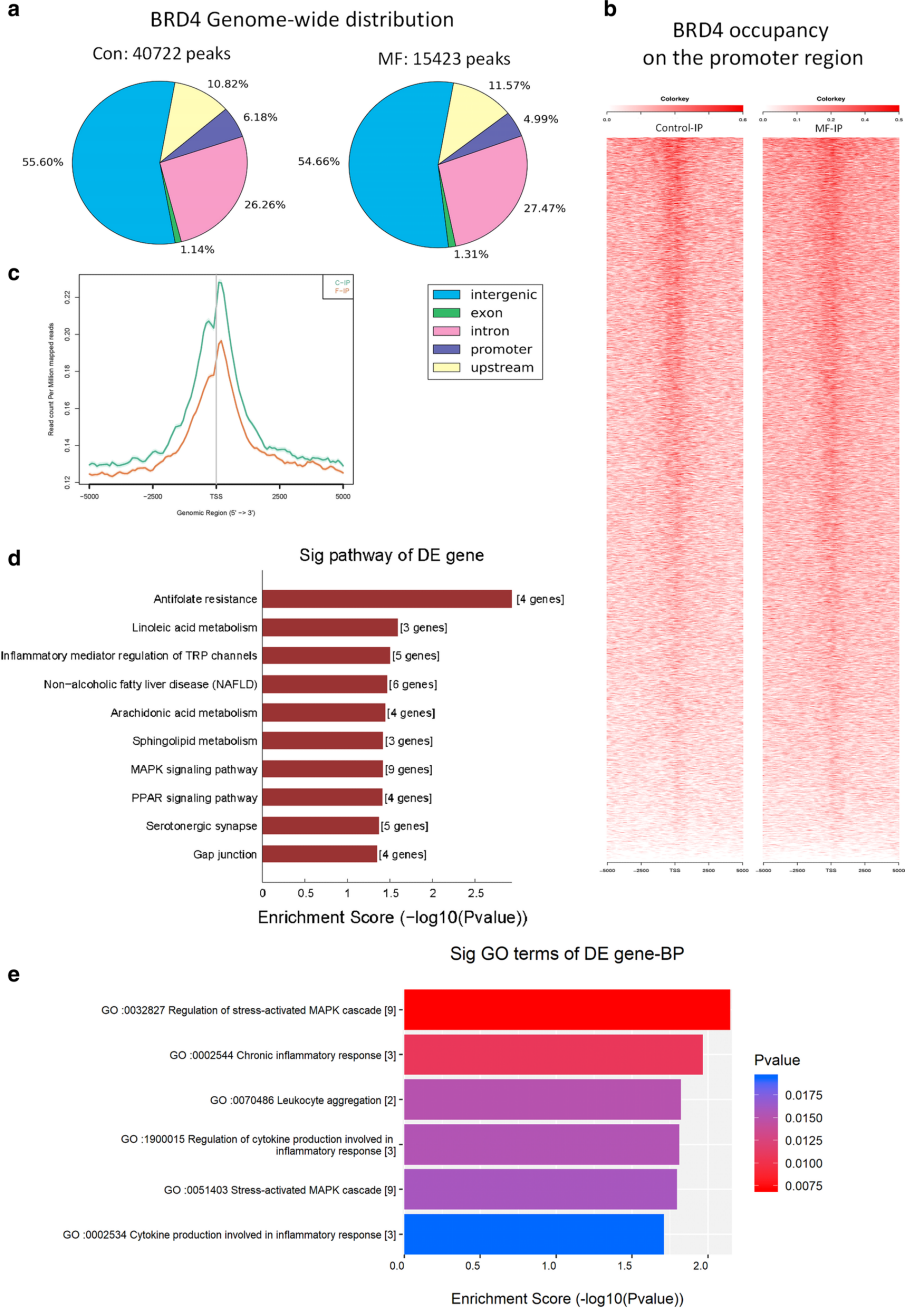
Overloading mechanical stress increases BRD4 occupancy on inflammation and stress-related genes promoter region. **a** Genome-wide distribution of BRD4 in condylar cartilage from control (Con) and mechanical force (MF) group. The numbers on the top represent total peak numbers. **b** Heat maps of BRD4 occupancy on the promoter region (TSS ± 5 kb), aligned by the degree of BRD4 signal intensity in condylar cartilage from control (Con) and mechanical force (MF) group. **c** ChIP-seq tag distribution of BRD4 surrounding the TSS (± 5 kb) of the whole genome in the condyles from Con and MF groups. **d** KEGG pathway analysis of increased BRD4-binding peaks in rat TMJ cartilage after overloading mechanical stress. The top ten most significant enrichment pathways and the related details were shown. **e** GO analysis of increased BRD4-binding peaks in rat TMJ cartilage after overloading mechanical stress. The top six most significant enrichment GO terms which are related to inflammation and the details were shown

ChIP-seq analysis for BRD4 revealed 40,722 peaks and 15,423 peaks in control and MF group, respectively (Fig. 3a). These peaks were distributed widely over the genic and intergenic regions. Approximately 45% of BRD4 peaks were found in genic regions, while the rest of BRD4 peaks (~ 55%) were distributed over the intergenic regions (Fig. 3a). There was increased BRD4 binding surrounding the transcription start site (TSS) at the genome-wide level between Con and MF groups (Fig. 3b, c). The KEGG pathway and GO analysis provided us some clues of BRD4’s regulation on inflammatory response. The top ten most significant enrichment pathways were obtained from the increased BRD4-binding peaks on the promoter region after overloading mechanical stress (Fig. 3d). Two inflammation-related pathways were included which were inflammatory mediator regulation of TRP channels (ranked 3rd) and MAPK signaling pathway (ranked 7th). Genes such as *Il-1β, Myc, Nf-κb* were involved (Additional file 1: Table S2) which may transcribe under mechanical stress and cause joint damage aggravating. The top four most significant enrichment GO terms which are related to inflammation are regulation of stress-activated MAPK cascade, chronic inflammatory response, leukocyte aggregation, regulation of cytokine production involved in inflammatory response (Fig. 3e). Genes included are listed in Additional file 1: Table S3.

### Excessive mechanical stress increases BRD4 binding to the H3K27ac on the promoter region of *Trem1* in vivo

**Fig. 4:**
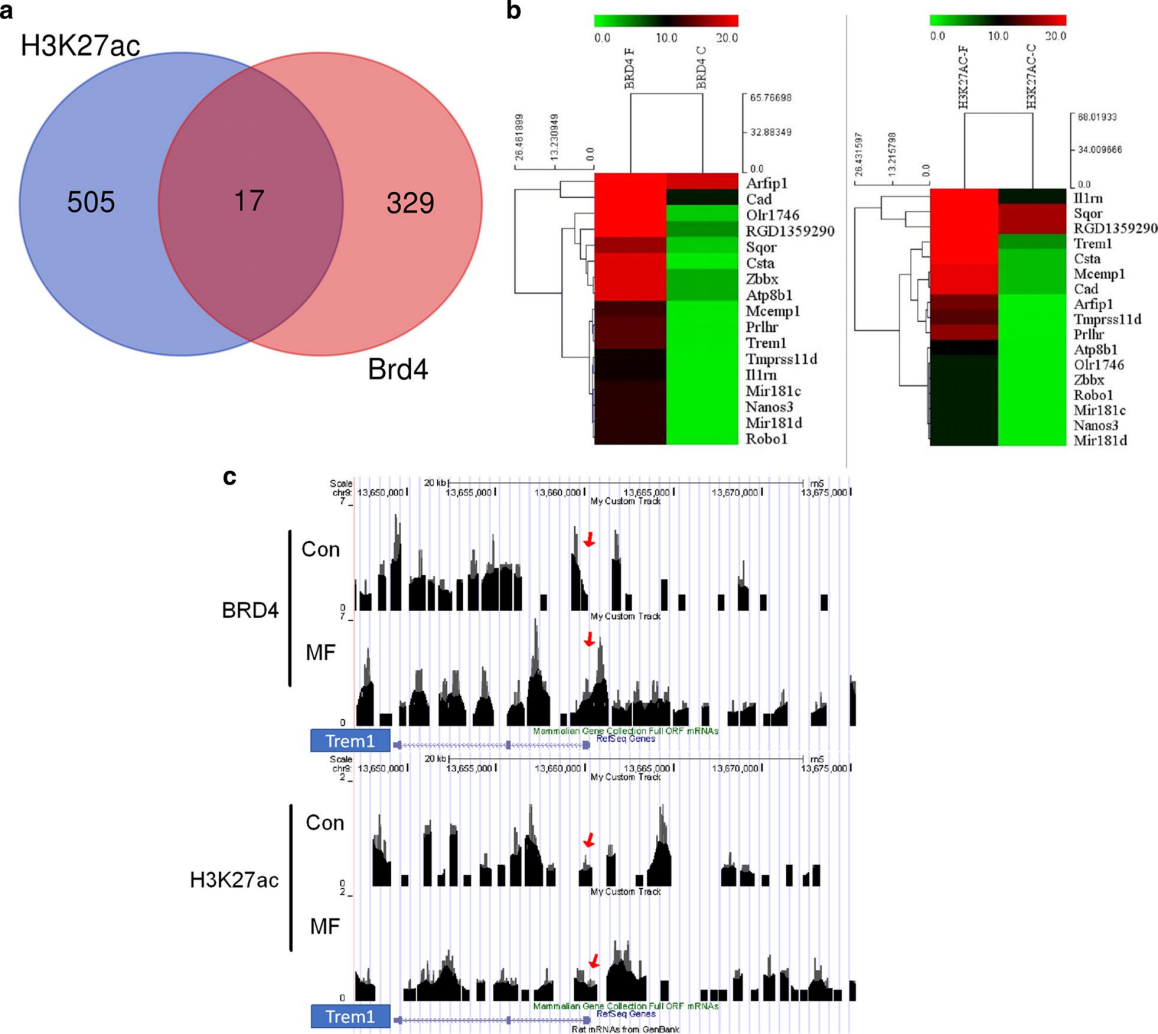
Overloading mechanical stress increases BRD4 binding to the H3K27ac on the promoter region of 17 genes including *Trem1*. **a** Venn diagram depicting the number of increased enriched genes binding to BRD4 or H3K27ac after overloading stress. **b** The 17 overlapped genes which have increased binding of BRD4 and H3K27ac were listed in the heat map. **c** Gene tracks of ChIP-seq signal for BRD4 and H3K27ac at the *Trem1* gene loci in control (top) and MF group (bottom). The y-axis shows the relative enrichment of the ChIP-seq signal. The x-axis depicts genomic position. Red arrow indicates the promoter region of *Trem1*

As BRD4 can recognize the H3K27ac site to regulate gene transcription [31–33], we also performed ChIP-seq with H3K27ac antibody using cartilage samples from control and mechanical force groups. The aim is to identify the overlapped increased BRD4-binding and H3K27ac-binding sites in the chondrocytes after overloading mechanical stress and to further investigate the role of BRD4 in mechanical stress-induced TMJ pathological changes. In total, 522 genes were identified to have increasing H3K27ac binding and 346 genes were identified to have increasing BRD4 binding in the promoter region after overloading mechanical stress (Fig. 4a). The 17 overlapped genes which have increased binding of BRD4 or H3K27ac were listed in the heat map (Fig. 4b). The details of the 17 genes are listed in Additional file 1: Table S4.

Through literature review, we found 4 genes are related to bone or joint diseases which are *Cystatin A*, *Mir-181d, Mir-181c*, and *Trem1*. Cystatin A (CSTA) and desmoplakin (DSP) interaction was found in human osteoarthritis, but the mechanism is unclear [34]. *Mir-181d* and *Mir-181c* are two miRNAs which are related to bone metabolism. *Mir-181d* can inhibit osteogenic differentiation of hBMSCs [35]. *Mir-181c* is the potential biomarkers for prognosis and diagnosis of osteoporosis [36]. *Trem1*, triggering receptor expressed on myeloid cells 1, was found to play a role in the regulation of inflammation. It was strongly upregulated in inflamed compared with normal/reactive areas of osteoarthritis synovial membrane, and it plays a critical in OA development through regulation of NF-κB signaling [29, 37, 38]. Gene tracks showed that overloading mechanical stress can increase BRD4 and H3K27ac co-occupancy on the promoter region of *Trem1* (Fig. 4c). As we focused on the anti-inflammatory effect of BRD4 inhibition and *Trem1* is a pro-inflammation factors with increased BRD4 binding after overloading, we hypothesized that BRD4-regulated inflammatory response may be mediated by *Trem1*.

### BRD4 inhibition reduces expression of inflammatory factors via regulating *Trem1*

**Fig. 5:**
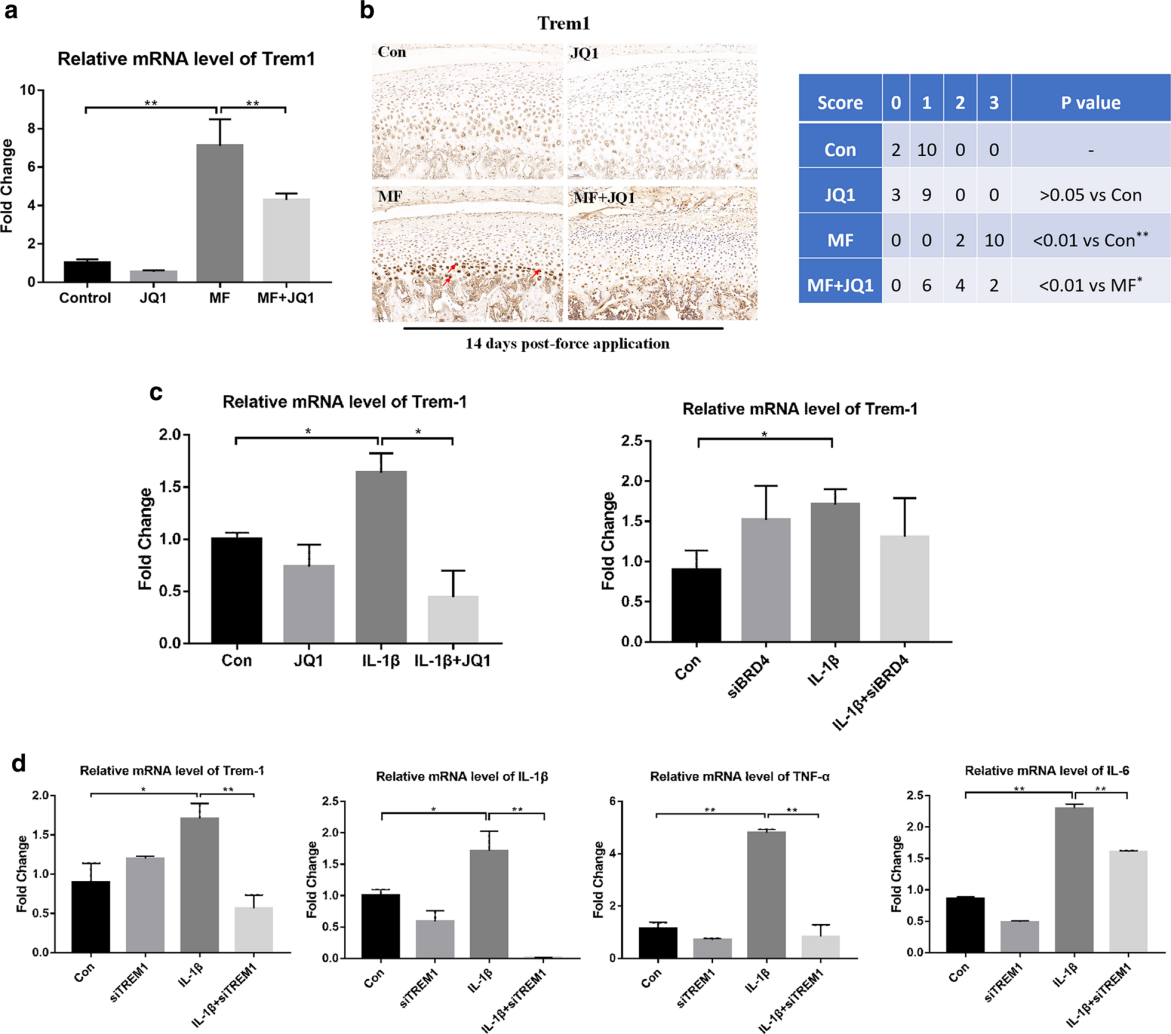
BRD4 inhibition reduces TREM1 induced by mechanical stress and IL-1β. TREM1 inhibition reduces inflammatory factors induced by IL-1β. **a** qRT-PCR analysis of *Trem1* expression in condylar cartilage at 14 days (*n* = 6). **b** Immunohistochemical analysis of TREM1 in condylar cartilage (200×) at 14 days (*n* = 12). TREM1 expressing chondrocytes are denoted (arrow). The sections were scored according to the intensity of the stain in the condyle (0, negative, no staining; 1, faint yellow, mild staining; 2, clay bank, moderate staining; and 3, brown, intense staining). The chondrocytes were treated with IL-1β (10 ng/mL) for 24 h. The cells were concurrently treated with JQ1 (400 nM) or siBRD4. **c** qRT-PCR analysis of *Trem1* in chondrocytes from samples treated as indicated. **d** qRT-PCR analysis of *Trem1, Il-1β, Tnf-α*, and *Il-6* in chondrocytes from samples treated as indicated. Error bars indicate the standard deviation (*P < 0.05; **P < 0.01)

It is found that both BRD4 inhibitor JQ1 and siBRD4 can decrease the expression of *Trem1* mRNA induced by the mechanical force in vivo or IL-1β in vitro (Fig. 5a–c). Next, we built the siTREM1 to investigate whether TREM1 can regulate other inflammatory factors (Additional file 1: Fig. S5). We found that in vitro IL-1β-induced increase of endogenous *Il-1β, Il-6*, and *Tnf-α* mRNA was reduced by siTREM1 (Fig. 5d). Combined with previous findings (Fig. 2), we concluded that BRD4 inhibition can regulate TREM1-mediated inflammatory response under mechanical stress or IL-1β treatment.

## Discussion

In our TMJ OA model induced by compressive stress, we found marked inflammatory cells infiltration in the subchondral bone near dilated blood vessels and increased inflammatory cytokines expression such as *Tnf-α, Il-1β, and Il-6*. Furthermore, we demonstrated that BRD4-mediated inflammatory responses play an important role in TMJ OA for the first time. Recent experimental data have shown that subchondral bone may have a substantial role in the OA process, as a source of inflammatory mediators implicated in the OA pain process and in the degradation of the deep layer of cartilage [39]. Clinical studies also demonstrated a direct association between joint inflammation and the progression of OA [2, 39]. Thereby, our results demonstrated that the inflammation is an important pathological change that cannot be ignored and targeting joint inflammation will help to slow the progression of TMJ OA. BRD4 inhibition such as JQ1 can be a potential therapeutic agent for TMJ OA.

In this study, to explore the mechanism under the protective effect of BRD4 inhibition on TMJ under mechanical stress, we used ChIP-seq to conduct high-throughput screening. With this technology, we can fully uncover the changes of BRD4’s binding to DNA under excessive mechanical stress. The BRD4 peaks decreased after excessive mechanical stress loading which means the number of BRD4’s binding sites decreased at the genome-wide level. But the KEGG pathway and GO analysis showed enriched BRD4 binding to several inflammation-related pathways and biological process which means BRD4 may binds more intensively to specific DNA region. Two inflammation-related pathways are screened out from BRD4 ChIP-seq analysis which are MAPK signaling pathway and inflammatory mediator regulation of TRP channels. The excessive mechanical stress can also increase BRD4 occupancy on the other inflammation-related genes which are involved in chronic inflammatory response, leukocyte aggregation, and regulation of cytokine production involved in inflammatory response. Our results showed that BRD4 could play a comprehensive role in regulating mechanical stress-induced TMJ inflammation.

**Fig. 6:**
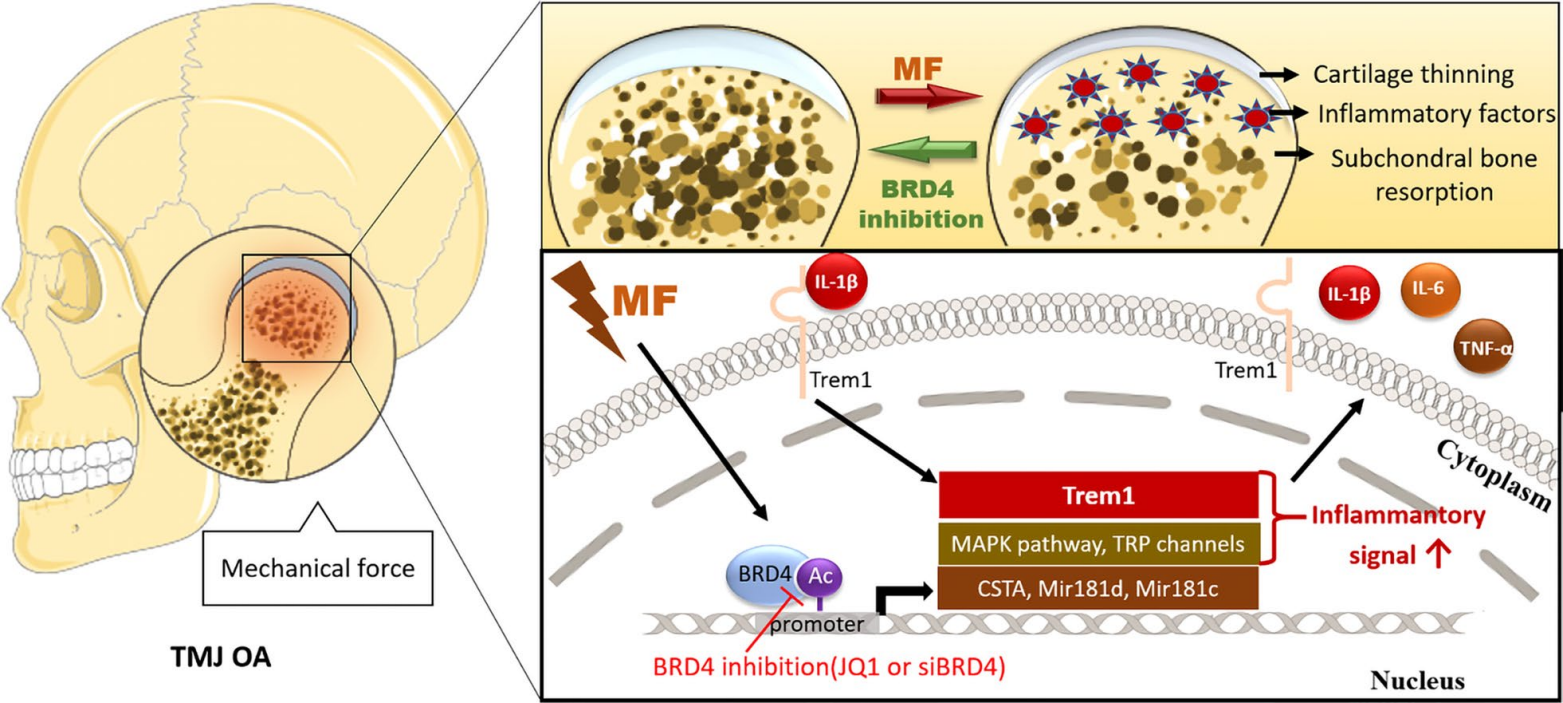
The role of BRD4 in the mechanical stress-induced TMJ OA pathological changes and inflammatory response of chondrocytes. The excessive mechanical stress is an important etiological factor of TMJ OA. It can cause cartilage thinning, subchondral bone destruction, and joint inflammation. BRD4 inhibition can alleviate the TMJ pathological changes induced by mechanical stress. The underlying mechanism is shown in the schematic diagram of the chondrocyte. At the cellular level, the binding of BRD4 to the promoter region of the inflammation-related genes and microRNA increased under mechanical stress stimulation. We confirmed the binding enhanced the transcription of *Trem1* and inflammatory factors such as *Il-1β, Tnf-α*, and *Il-6*. After these receptor and inflammatory factors are expressed, they aggravated the inflammatory response of the chondrocytes. IL-1β-induced inflammation is TREM1-dependent, and both IL-1β and TREM1 are regulated by BRD4. Our study indicates that BRD4 plays an important role in mechanical stress-induced TMJ pathological changes and especially cartilage inflammatory response. TREM1 is involved in the BRD4’s epigenetic regulation of inflammation

*Trem1* was screened out from BRD4 and H3K27ac ChIP-seq. TREM1 is an immunoglobulin-like cell surface receptor first found in polymorphonuclear cells [40]. Its activation leads to the NF-κB signaling pathway activation and the production of multiple proinflammatory cytokines and chemokines [41, 42]. Dan reported that TREM1 is a therapeutic target to inhibit the inflammatory response in rheumatoid arthritis [38]. Tang reported that knockdown of *Trem1* suppresses IL-1β-induced chondrocyte injury [29]. Clinical study showed TREM1 was strongly upregulated in the inflamed synovial membrane [37] and cartilage [29] from TMJ OA patients. In this study, we found excessive mechanical stress can increase BRD4 and H3K27ac co-occupancy on the promoter region of *Trem1* and increase TREM1 expression. We furthermore confirmed BRD4 can regulate TREM1 expression induced by both mechanical force or IL-1β and TREM1’s ability to regulate inflammation. Our results first revealed the relation between BRD4 and TREM1 and proved that BRD4 inhibition alleviates TMJ inflammation under mechanical stress partly via regulating TREM1 (Fig. 6).

The limitation of this study is that we did not prove TREM1’s role in mechanical stress-induced TMJ OA in vivo. Although we confirmed the BRD4 can regulate TREM1 and we performed in vitro study to prove TREM1 can regulate downstream inflammatory genes expression under IL-1β treatment, we are still not ensured whether TREM1 plays an important role in the pathological changes of TMJ under mechanical stress loading. If we can confirm that, the role of BRD4-TREM1 axis in TMJ OA can be more convinced. Whether other screened out genes and pathways anticipate in the protective effect of BRD4 inhibition on TMJ OA also needs further research.

## Conclusions

In summary, this study for the first time focused on the effects of BRD4 inhibition on excessive mechanical stress-induced TMJ pathological changes and explored the mechanism. We concluded that BRD4 inhibition can protect TMJ from cartilage thinning and subchondral bone resorption and exerts an anti-inflammatory effect via regulating TREM1 under mechanical stress. Our findings highlight the protective effect of BRD4 inhibition on TMJ OA and suggest that JQ1 might be a novel therapeutic agent against TMJ OA.

## Materials and methods

### Animal models

Experimental protocols were reviewed and approved by the Animal Care and Use Committee of Nanjing University. This study also complied with Animal Research: Reporting In Vivo Experiments (ARRIVE) guidelines for preclinical animal studies. All animals were housed in an approved facility at Nanjing University. One hundred and sixty-eight condyles from eighty-four 8-wk-old male Sprague–Dawley rats were used in this study. The animals were housed in a light- and temperature-controlled room and given unrestricted access to food and water during the experimental period. Before the start of the experiment, all of the animals were acclimated to their surroundings for 1 day. All of the animals were randomly divided into non-mechanical force (Control) group and mechanical force (MF) group. All of the rats in the MF groups were loaded with 80 g compressive mechanical force on the first day of the experiment as previously described [6, 43]. Briefly, 2 hooks were made with 0.025-inch stainless steel wire after the lower incisors of the rats were cleaned; then, resin was shaped into a sphere so that an undercut was formed and the hooks could not come out. The anchorage jigs were made of copper wires and were placed around the neck and arms. A rubber band was tied between the anchorage jig and the hook on the same side to load 80 g of compressive mechanical force upward and backward on each side (Additional file 1: Fig. S2). The rats in MF group wore the loading appliance for 14 d. None of the rats displayed signs of disability, and all of the animals received the same standardized diet throughout the procedure. (+/−)-JQ1(SML0974, Sigma) (50 μM) was injected locally into the temporomandibular joint (TMJ) on one side, and vehicle (dimethyl sulfoxide; Solarbio) was injected on the other side as described [44] at the 4th, 7th, and 11th day after mechanical force loading. A tailored micro-injector was inserted just below the zygomatic arch between the eye and ear until the outer surface of the mandibular ramus was reached. The orientation of the needle head was adjusted to enable the needle to pass along the bone wall and finally reach the TMJ region.

### Tissue preparation and histological staining

All of the animals and their age-matched controls were killed at the 14th day by cervical dislocation under diethyl ether anesthesia. TMJ specimens were harvested. These specimens were fixed in formalin for 24 h and decalcified in ethylenediaminetetraacetic acid (EDTA) for 8 weeks at 4 °C. Then, they were dehydrated, embedded in paraffin, and sectioned through the TMJ sagittal plane to make 5-μm-thick serial sections. The paraffin sections were deparaffinized in xylene and rehydrated through graded alcohols. Then, 3 slides of central sagittal sections from each sample were stained with hematoxylin and eosin (HE) for histological assessment. The thickness of the mandibular condylar cartilage and number of osteoclasts were measured by computerized morphometry, which was carried out by a single observer who was blinded to the experimental protocol. All images were captured by an Olympus XI 70 microscope equipped with an Olympus Magna Fire digital camera and were analyzed using a computerized digital image analysis system (Image-Pro Plus, version 6.0; MediaCybernetics). The condylar cartilage was equally divided into 3 parts: the anterior, middle, and posterior thirds, as previously described [6]. We mainly focused on changes in the middle third, which is the main load-bearing area based on the direction of force application (Additional file 1: Fig. S3). The thickness of the cartilage was measured in 3 squares located at the quartering points of the center third of the cartilage [45]. The mean of the values for the 3 squares in each section was used for further statistical analysis.

### Immunohistochemistry

Immunohistochemistry was performed as previously described [6]. In brief, 5-μm-thick sections (3 sections per specimen) were prepared according to standard protocols for HE staining. Rabbit anti-IL-1β (sc-7884, Santa Cruz, USA), Rabbit anti-TNF-α (ab6671, Abcam, Hong Kong), and Rabbit anti-TREM1 (AF8226, Beyotime, Shanghai) served as the primary antibodies, and goat anti-rabbit horseradish peroxidase–conjugated IgG served as the secondary antibody. The slides were incubated with primary antibodies overnight at 4 °C and with secondary antibodies for 30 min at 37 °C. Diaminobenzidine (SP immunochemistry kit; Boster Biological Technology, Ltd.) was then applied, followed by counterstaining with hematoxylin. Image acquisition was performed using the same method used for the HE-stained sections. The immunohistochemical measurements were taken by 2 observers. The sections were scored according to the intensity of the stain in the condyle (0, negative, no staining; 1, faint yellow, mild staining; 2, clay bank, moderate staining; and 3, brown, intense staining). Interobserver differences were reconciled through microscope conferencing.

### Micro-CT analysis

Routine calibration was performed once per week using a three-point calibration phantom corresponding to a density range from air to cortical bone. The rats TMJs were scanned with an ex vivo micro-CT system (GE eXplore Locus SP, London) at 80 kV and 80 mA. The X-ray image was reconstructed with an isotropic voxel size of 9 μm. After acquiring the radiographic data, images were processed using the Health Care MicroView ABA software. The value of BV/TV, Tb.N, Tb.Th, and Tb.Sp was measured.

### Mandibular chondrocyte culture

Ninety-six mandibular condylar cartilage was isolated from forty-eight 3-wk-old male Sprague–Dawley rats and subsequently minced. After the primary condylar chondrocytes were digested with 0.25% trypsin (Gibco/Invitrogen) for 30 min and with 0.2% type II collagenase (Sigma-Aldrich) for 3 h, they were rinsed 3 times and prepared as a single cell suspension. The primary condylar chondrocytes were counted with a hemocytometer. The chondrocytes were seeded at a density of 1 × 10^5^ cells/cm^2^ in a humidified atmosphere of 37 °C and 5% CO_2_. The cells were cultured to the third generation for subsequent experiments. The cultured cells were divided into 4 groups according to the treatment they received. These groups included the control, JQ1 (cells treated with 400 nM), IL-1β (cells treated with 10 ng/mL IL-1β), IL-1β + JQ1 (cells treated concurrently with 10 ng/mL IL-1β and 400 nM JQ1).

### Small Interference RNA (siRNA) infection

Small interfering RNA (siRNA) targeting BRD4 (siBRD4), small interfering RNA (siRNA) targeting TREM1 (siTREM1), and non‐silencing siRNA were transformed into RNA oligos (siRNA), respectively. The BRD4 silencing sequences were 5′- GAACCUCCCUGAUUACUAUTTAUAGUAAUCAGGGAGGUUCTT-3′. The TREM1 silencing sequences were 5′-GACAGACUCUGGAUUAUAUTTA UAUAAUCCAGAGUCUGUCTT-3′. The non‐silencing siRNA was sense-5′-UUCU CCGAACGUGUCACGUTT-3 & antisense-5′-ACGUGACACGUUCGGAGAATT-3′. Lipofectamine 2000 reagent (Invitrogen, Carlsbad, CA, USA) was inserted into the mixing solution for 20 min, and chondrocytes were harvested after 48 h.

### Isolation of total RNA and qRT-PCR

As the condyle is very small, we used three condyles from three different rats as a single cartilage sample for RNA extraction in the in vivo model. In the in vitro model, different groups of cells from a 6-well plate were collected. Total RNA was extracted using TRIzol (Invitrogen Life Technologies, CA, USA). The primers used are listed in Additional file 1: Table S1. All the genes were analyzed using a 7500 Real-Time PCR machine (Applied Biosystems, CA, USA) using glyceraldehyde-3-phosphate dehydrogenase (GAPDH) as an internal control. The results were analyzed as the relative quantification compared to the control group. Each experiment was conducted three times, and the average values were calculated.

### ChIP sequencing

ChIP sequencing was performed on condylar cartilage samples from control and mechanical force group using BRD4 antibody (39,909, ACTIVE MOTIF, America) and H3K27ac antibody (ab4729, Abcam, the USA) by KangChen (KangChen Bio-tech Inc, Shanghai, China). The detailed method and results are available from the corresponding author upon reasonable request. The purity and concentration of DNA samples were determined with Qubit® Fluorometer. In total, 10 ng of DNA samples was prepared for Illumina sequencing. Size selection of ~ 200–1500 bp enriched product using AMPure XP beads. The completed libraries were quantified by Agilent 2100 Bioanalyzer. The libraries were denatured to generate single-stranded DNA molecules and sequenced on the Illumina HiSeq 4000 following the HiSeq 3000/4000 SBS Kit (300 cycles) protocol. After the sequencing platform generated the sequencing images, the stages of image analysis and base calling were performed using Off-Line Basecaller software (OLB V1.8). Sequence quality was examined using the FastQC software. After passing Solexa CHASTITY quality filter, the clean reads were aligned to Rat reference genome RN5 using BOWTIE (V2.1.0). Aligned reads were used for peak calling of the ChIP regions using MACS V1.4.2. Statistically significant ChIP-enriched regions (peaks) were identified by comparison of IP vs input or comparison to a Poisson background model, using a p value threshold of 10^−4^. Then the peaks were annotated by the nearest gene using the newest UCSC RefSeq database. To generate heat maps for BRD4 occupancy on promoters, BRD4 signals were displayed on the promoter regions (encompassing the TSS ± 5 kb), from condyles in Con and MF groups. Rows are ranked by BRD4 signal intensity. The density of BRD4 ChIP-seq signals was determined for TSS ± 5 kb region shown as the read count per million mapped reads. GO analysis and pathway analysis were performed using R language package (R 3.5.0). ChIP-seq profile was visualized using UCSC Genome Browser (http://genome.ucsc.edu/cgi-bin/hgGateway).

### Statistical analysis

All measurements were repeated three times. The data are expressed as means and standard deviations. All statistical analyses were conducted using the two-tailed, unpaired t test (two conditions) or one-way ANOVA. All data were analyzed with SPSS 16.0 software, and P < 0.05 was used to define statistical significance.

## Supplementary information

**Additional file 1: Figure S1.** Detailed description and diagram of the in vivo and in vitro experimental design. **Figure S2.** Establishment of the TMJ-OA rat model. **Figure S3.** Anterior, middle, and posterior thirds of the condylar cartilage. **Figure S4.** BRD4 expression in normal articular chondrocytes infected with siBRD4, compared with negative control. **Figure S5.** TREM1 expression in normal articular chondrocytes infected with siTREM1, compared with negative control. **Figure S6.** Condylar cartilage thinning induced by mechanical stress at 4d, 7d and 14d. **Figure S7.** BET inhibitor JQ1(5μM, 10μM, 50μM) can relieve the condylar cartilage thinning and reduce the expression of inflammatory factors induced by overloading mechanical stress. **Figure S8.** The Survival rate of condylar chondrocytes of rats treated with JQ1 and IL-1β of different concentration. **Table S1.** Primers Used in Real-Time RT-PCR. **Table S2.** KEGG pathway analysis of increased BRD4-binding peaks in rat TMJ cartilage after overloading mechanical stress. **Table S3.** GO analysis of increased BRD4-binding peaks in rat TMJ cartilage after overloading mechanical stress. **Table S4.** The details of 17 genes with increased BRD4 and H3K27ac overlapped bindings in the promoter region after overloading mechanical force.

## Data Availability

The datasets used and/or analyzed during the current study are available from the corresponding author on reasonable request (lihuang76@nju.edu.cn).

## Abbreviations

TMJ OA: Temporomandibular joint osteoarthritis
siRNA: Small interference RNA
ECM: Extracellular matrix
ARRIVE: Animal Research: Reporting In Vivo Experiments
MF: Mechanical force group
Control: Control group
EDTA: Ethylenediaminetetraacetic acid
HE: Hematoxylin and eosin
BV/TV: The ratio of bone volume to tissue volume
Tb.N: Trabecular number
Tb.Th: Trabecular thickness
Tb.Sp: Trabecular separation
FP: Fibrous proliferative layer
T: Transition layer
H: Hypertrophic layer
TSS: Transcription start site
CSTA: Cystatin A
DSP: Desmoplakin
