# Supplementary material for: BRD4 inhibition alleviates mechanical stress-induced TMJ OA-like pathological changes and attenuates TREM1-mediated inflammatory response
Source: Clin Epigenetics. 2021 Jan 15;13:10. doi: 10.1186/s13148-021-01008-6 (PMC7809762; doi:10.1186/s13148-021-01008-6)
Supplement: Supplementary file 1 — Additional file 1: Figure S1. Detailed description and diagram of the in vivo and in vitro experimental design. Figure S2. Establishment of the TMJ-OA rat model. Figure S3. Anterior, middle, and posterior thirds of the condylar cartilage. Figure S4. BRD4 expression in normal articular chondrocytes infected with siBRD4, compared with negative control. Figure S5. TREM1 expression in normal articular chondrocytes infected with siTREM1, compared with negative control. Figure S6. Condylar cartilage thinning induced by mechanical stress at 4d, 7d and 14d. Figure S7. BET inhibitor JQ1(5μM, 10μM, 50μM) can relieve the condylar cartilage thinning and reduce the expression of inflammatory factors induced by overloading mechanical stress. Figure S8. The Survival rate of condylar chondrocytes of rats treated with JQ1 and IL-1β of different concentration. Table S1. Primers Used in Real-Time RT-PCR. Table S2. KEGG pathway analysis of increased BRD4-binding peaks in rat TMJ cartilage after overloading mechanical stress. Table S3. GO analysis of increased BRD4-binding peaks in rat TMJ cartilage after overloading mechanical stress. Table S4. The details of 17 genes with increased BRD4 and H3K27ac overlapped bindings in the promoter region after overloading mechanical force. [file 13148_2021_1008_MOESM1_ESM.docx]

**Content**

1. Figure S1 Detailed description and diagram of the in vivo and in vitro experimental design
2. Figure S2 Establishment of the TMJ-OA rat model
3. Figure S3 Anterior, middle, and posterior thirds of the condylar cartilage
4. Figure S4 BRD4 expression in normal articular chondrocytes infected with siBRD4, compared with negative control
5. Figure S5 TREM1 expression in normal articular chondrocytes infected with siTREM1, compared with negative control
6. Figure S6 Condylar cartilage thinning induced by mechanical stress at 4d, 7d and 14d
7. Figure S7 BET inhibitor JQ1(5μM, 10μM, 50μM) can relieve the condylar cartilage thinning and reduce the expression of inflammatory factors induced by overloading mechanical stress.
8. Figure S8 The Survival rate of condylar chondrocytes of rats treated with JQ1 and IL-1β of different concentration.
9. Table S1 Primers Used in Real-Time RT-PCR
10. Table S2. KEGG pathway analysis of increased BRD4-binding peaks in rat TMJ cartilage after overloading mechanical stress.
11. Table S3. GO analysis of increased BRD4-binding peaks in rat TMJ cartilage after overloading mechanical stress.
12. Table S4. The details of 17 genes with increased BRD4 and H3K27ac overlapped bindings in the promoter region after overloading mechanical force.

**A**


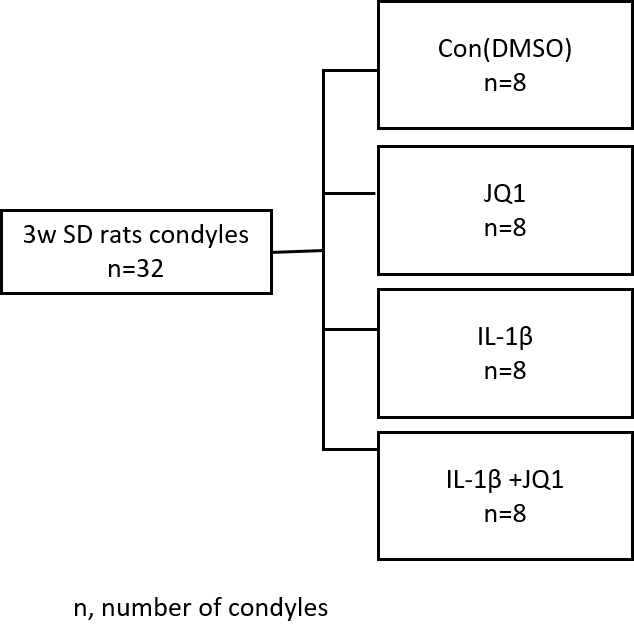

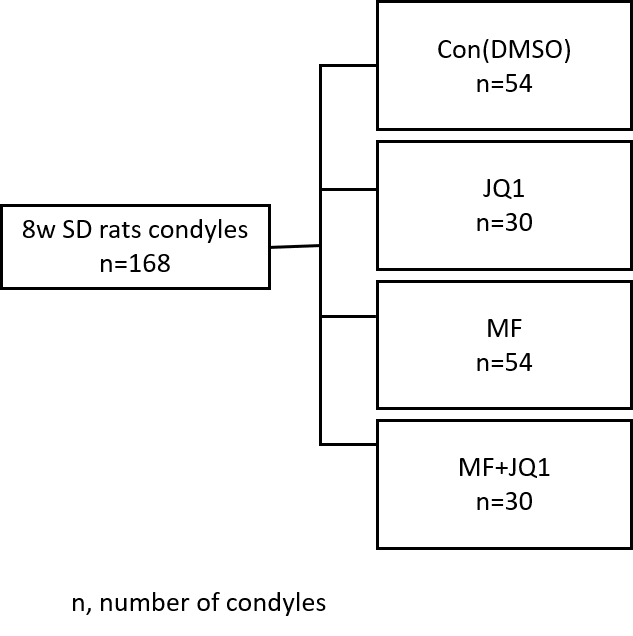

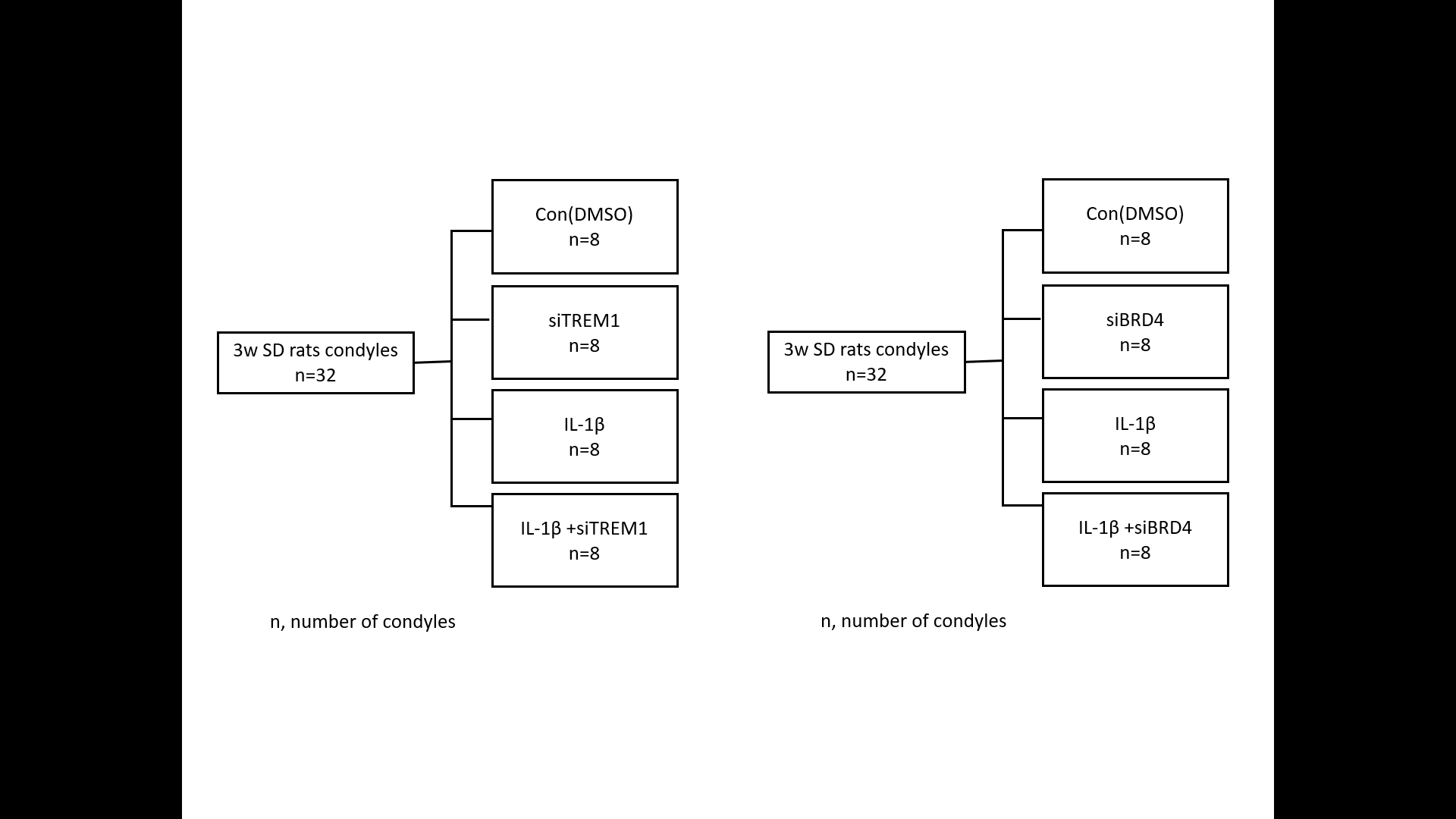


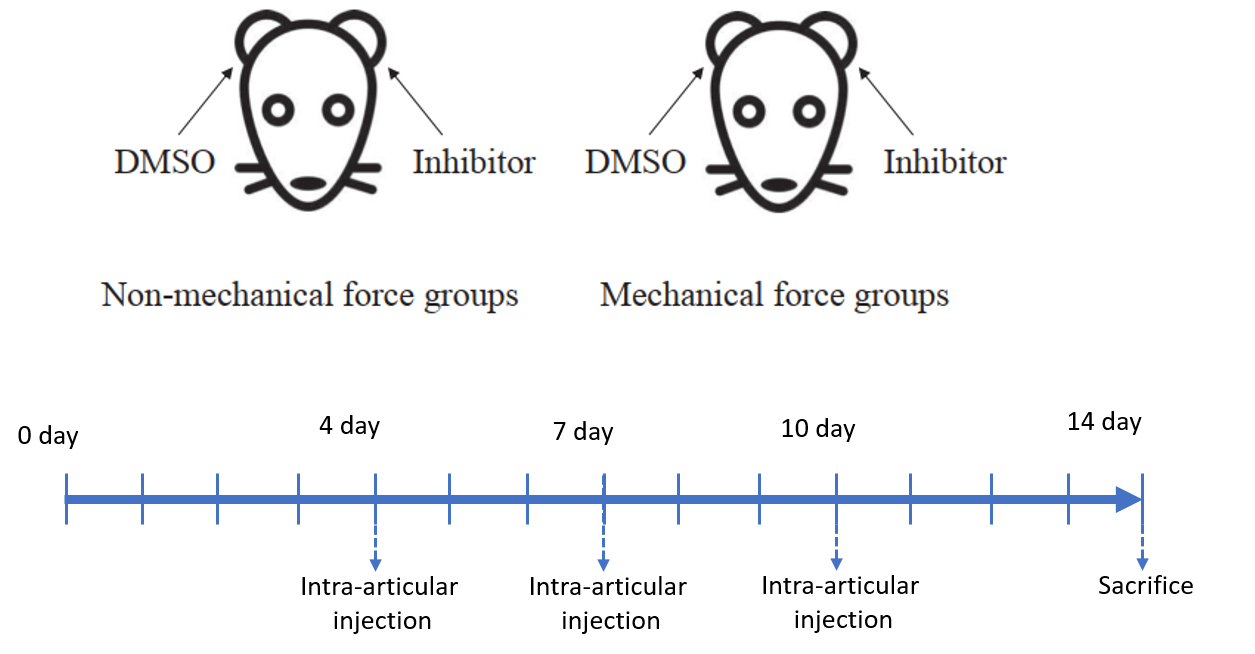


Con

MF+JQ1

JQ1

MF

**B**

Additional file 1: Fig. S1. (A) Detailed description of the in vivo (8w SD rat) and in vitro (3w SD rat) experiment. (B) Diagram of the in vivo experimental design.


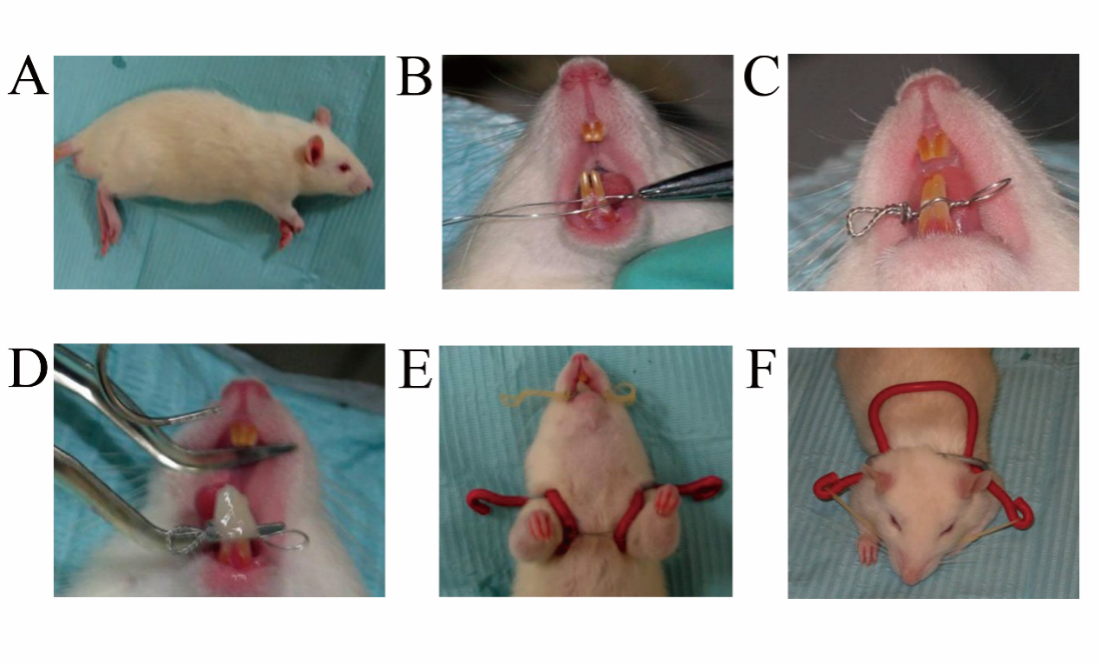


Additional file 1: Fig S2. Establishment of the TMJ-OA rat model. (A) Rats were anesthetized with chloral hydrate by intraperitoneal injection. (B, C) Two traction ring was made with 0.25inch ligation wire and (D) was fixed with 3M orthodontic bonding resin. (E) The anchorage jigs were made of copper wires and were placed around the neck and arms. (F) Rubber bands was tied between the anchorage jig and the hook to load 80 g of compressive mechanical force upward and backward on each side.


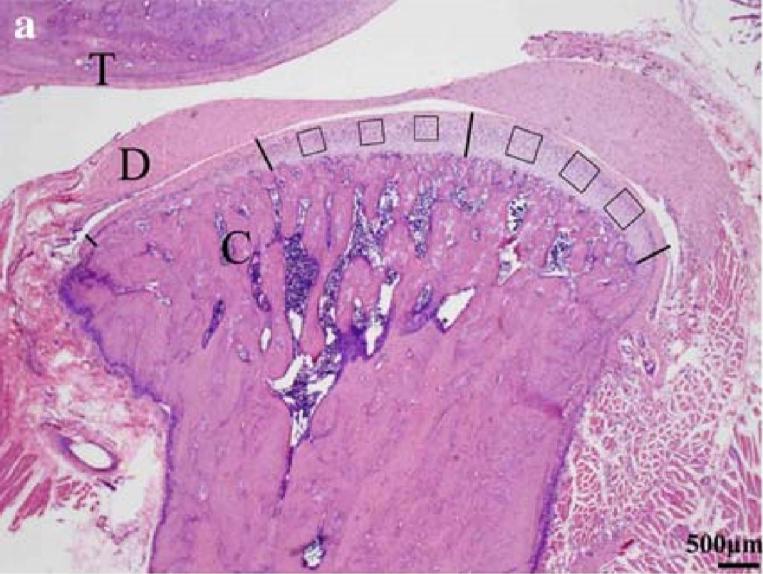


Additional file 1: Fig S3. Anterior, middle, and posterior thirds of the condylar cartilage. (PMID: 19052875) (T) temporal bone (D) articular disc (C) mandibular condyle. The mandibular condylar cartilage was equally divided into three parts: the anterior, middle, and posterior thirds. The middle third, which is the main load-bearing area based on the direction of force application. The 3 rectangles in the middle third of the cartilage illustrate the 3 regions (squares) within which we measured.


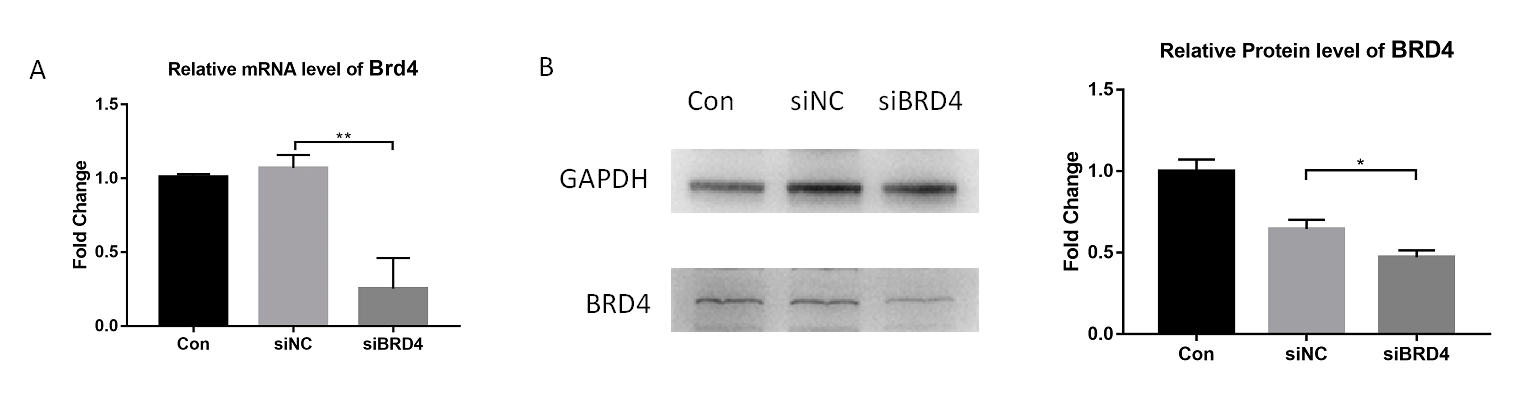


Additional file 1: Fig S4. BRD4 expression in normal articular chondrocytes infected with siBRD4, compared with negative control, using qRT-PCR (A) and western blot (B). (*P<0.05, **P<0.01).


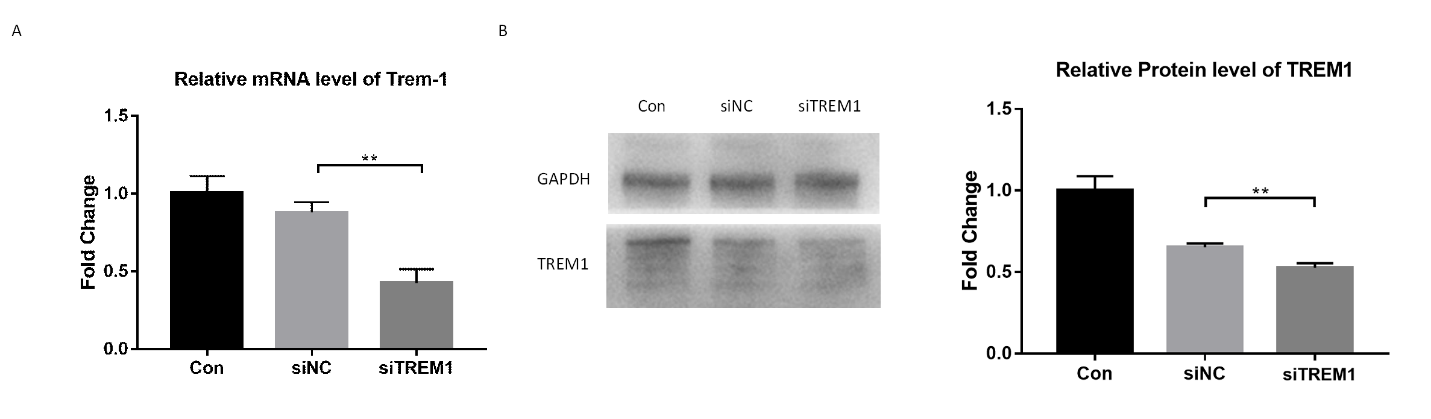


Additional file 1: Fig S5. TREM1 expression in normal articular chondrocytes infected with siTREM-1, compared with negative control, using qRT-PCR (A) and western blot (B). (*P<0.05, **P<0.01).


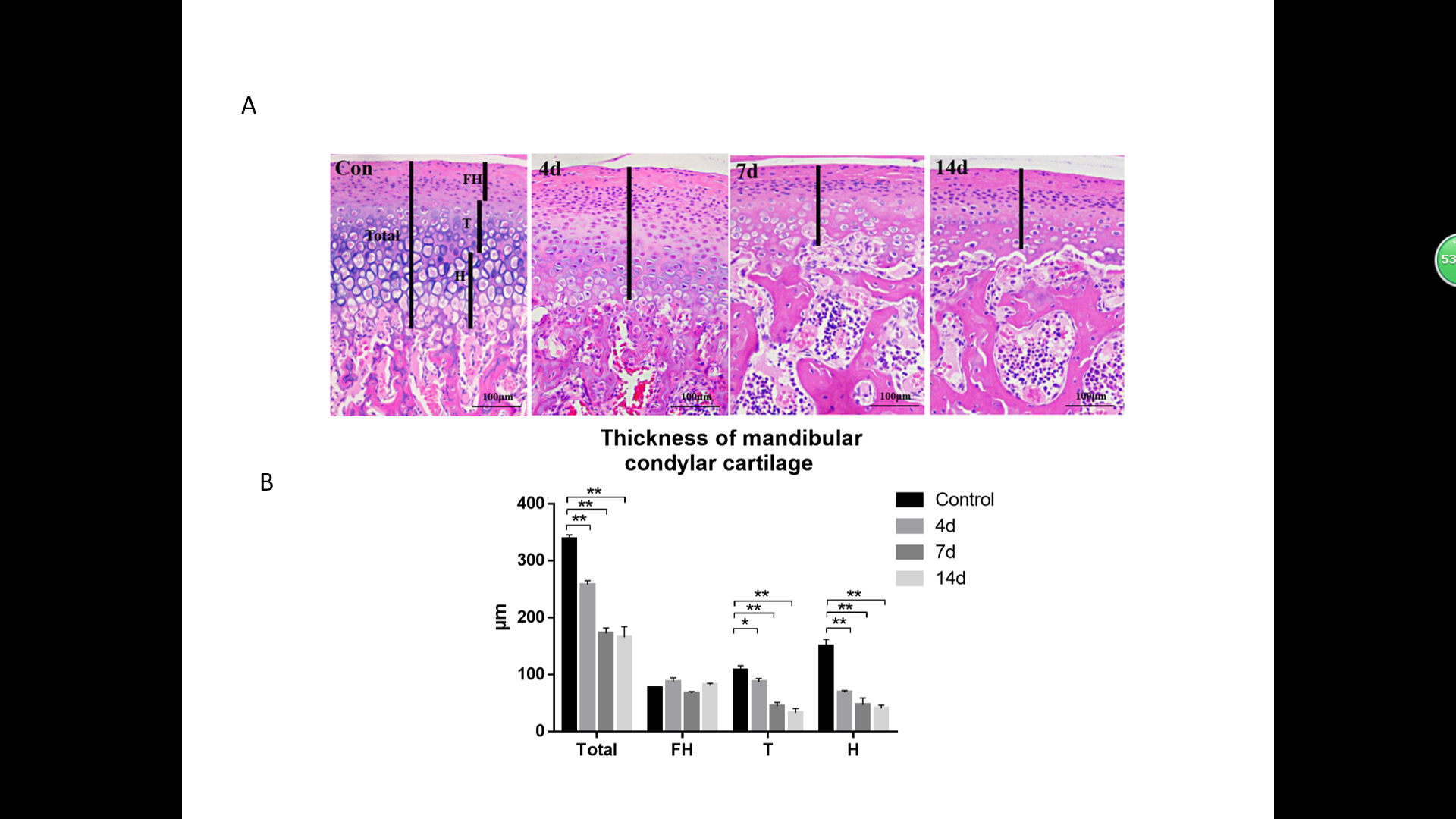


Additional file 1: Fig S6. Condylar cartilage thinning induced by overloading mechanical stress. (A) HE-stained sections (200×) of cartilage after force application for the number of days indicated (*n* = 12). Scale bar indicates 100 μm. Cartilage thickness is indicated (black bar). (B) Quantitative analysis of cartilage thickness of the samples shown in S6A. FH indicates proliferative zone, T indicates transition zone, and H indicates hypertrophic zone. (*n* = 12) Error bars indicate the standard deviation. (*P<0.05, **P<0.01). MF, mechanical force.


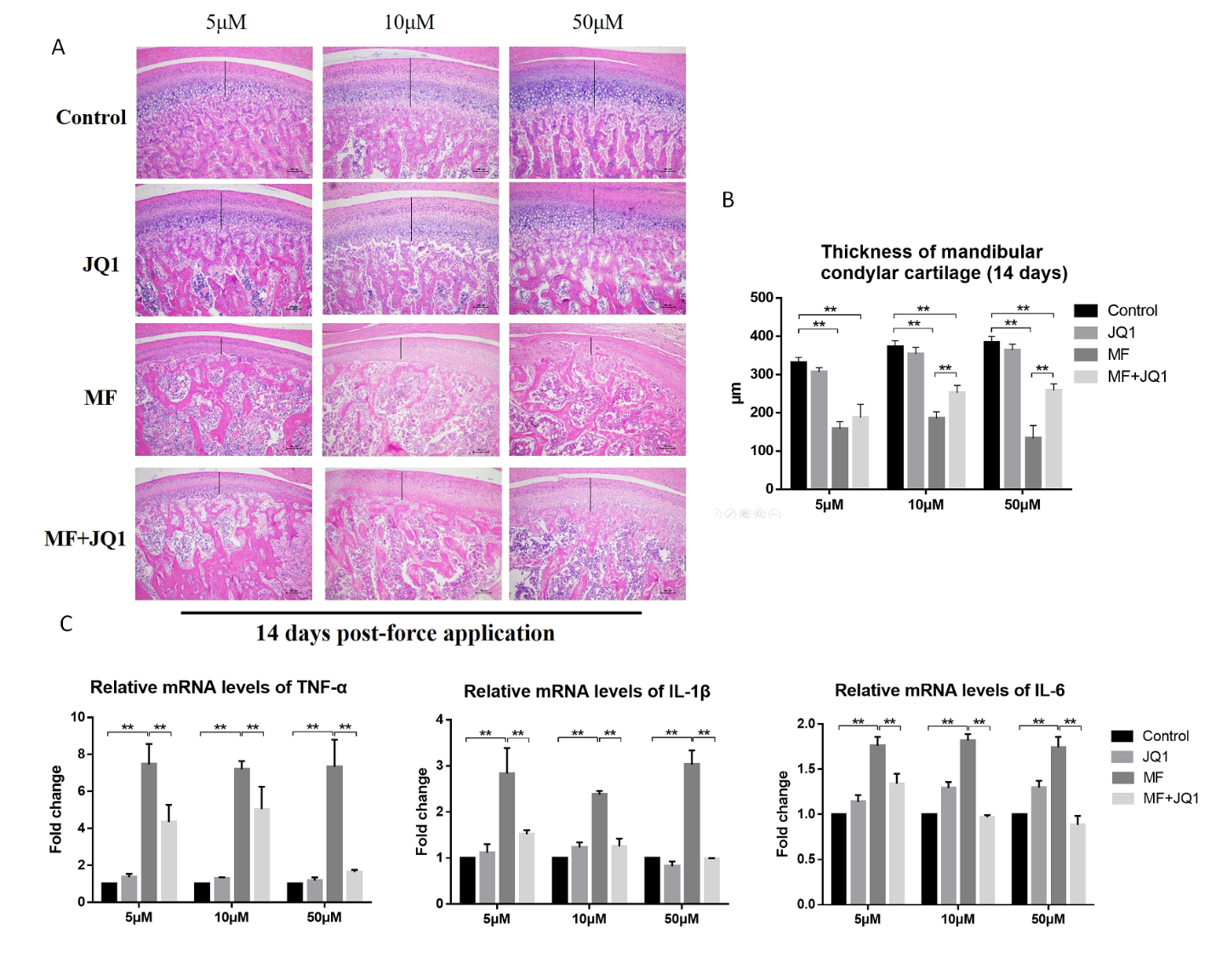


Additional file 1: Fig S7. BET inhibitor JQ1 can relieve the condylar cartilage thinning and reduce the expression of inflammatory factors induced by overloading mechanical stress. (A) HE-stained sections (100×) of cartilage treated as indicated at 14d with JQ1 in different concentration (*n* = 12). Scale bar indicates 100 μm. Cartilage thickness is indicated (black bar). (B) Quantification of cartilage thickness of the samples shown in S7A. (*n* = 12). (C) qPCR analysis of *Tnf-α, Il-1β* and *Il-6* expression in condylar cartilage treated as indicated (*n* = 12). Error bars indicate the standard deviation. (*P<0.05, **P<0.01). MF, mechanical force.


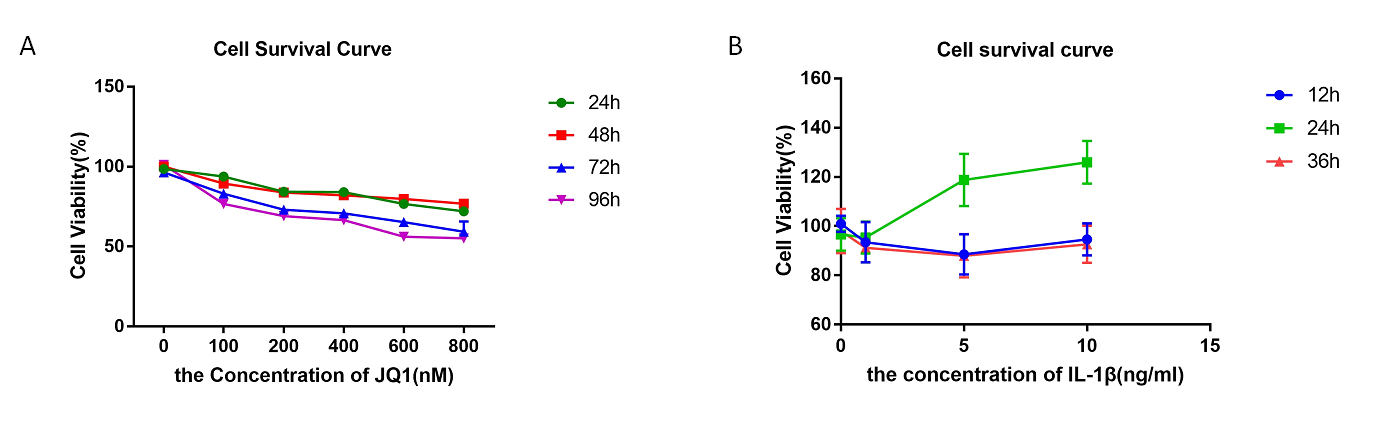


Additional file 1: Fig S8. The Survival rate of condylar chondrocytes of rats treated with JQ1 and IL-1β of different concentration. (A) The Survival rate of condylar chondrocytes of rats were treated with 0nM, 100nM, 200nM, 400nM, 600nM and 800nM JQ1 for 24h, 48h, 72h and 96h separately. (B) The survival rate of condylar chondrocytes of rats were treated with MTT assay after 12h, 24h, and 36h with IL-1β concentrated in 0ng/ml, 1ng/ml, 5ng/ml, and 10ng/ml separately.

Additional file 1: Table S1 Primers Used in Real-Time RT-PCR

| Gene | Primer sequences |
| --- | --- |
| *Gapdh* | Fwd-5'- GCAAGTTCAACGGCACAG -3'  Rev- 5'- CCAGTAGACTCCACGACAT -3’ |
| *Il-1β* | Fwd-5'-CCAGGATGAGGACCCAAGCA-3'  Rev- 5'-TCCCGACCATTGCTGTTTCC-3' |
| *Il-6* | Fwd-5'-GACAAAGCCAGAGTCATTCA-3'  Rev- 5'-TTGCCGAGTAGACCTCATAG-3' |
| *Tnf-α* | Fwd-5'-CCACGCTCTTCTGTCTACTG-3'  Rev- 5'-GCTACGGGCTTGTCACTCGA-3' |
| *Trem1* | Fwd- 5'-AAGGCTTGGCAGAGGCTATCA -3'  Rev- 5'-TAGGGTCATCTTTCAGGGTGTACT -3' |

Additional file 1: Table S2. KEGG pathway analysis of increased BRD4-binding peaks in rat TMJ cartilage after overloading mechanical stress.

| Definition | Fisher-P value | Selection Counts | Selection  Size | Count | Size | Enrichment Score | Gene Ratio | Genes |
| --- | --- | --- | --- | --- | --- | --- | --- | --- |
| Antifolate resistance - Rattus norvegicus (rat) | 0.001180603 | 4 | 134 | 31 | 8790 | 2.927896 | 0.029851 | *Abcg2//Alox12e//Il1b//Tyms* |
| Linoleic acid metabolism - Rattus norvegicus (rat) | 0.02580577 | 3 | 134 | 42 | 8790 | 1.588283 | 0.022388 | *Cyp2c22//Cyp2e1//Pla2g4b* |
| **Inflammatory mediator regulation of TRP** **channels - Rattus norvegicus (rat)** | 0.03196604 | 5 | 134 | 116 | 8790 | 1.495311 | 0.037313 | ***Alox12e//Cyp2c22//Htr2b//Il1b//Pla2g4b*** |
| Non-alcoholic fatty liver disease (NAFLD) - Rattus norvegicus (rat) | 0.03448873 | 6 | 134 | 159 | 8790 | 1.462323 | 0.044776 | *Cyp2e1//Il1b//Insr//Map3k11//Ndufa5//Ndufv1* |
| Arachidonic acid metabolism - Rattus norvegicus (rat) | 0.03626705 | 4 | 134 | 82 | 8790 | 1.440488 | 0.029851 | *Alox12e//Cyp2c22//Cyp2e1//Pla2g4b* |
| Sphingolipid metabolism - Rattus norvegicus (rat) | 0.03837064 | 3 | 134 | 49 | 8790 | 1.416001 | 0.022388 | *Cers5//Degs2//Neu3* |
| **MAPK signaling pathway - Rattus norvegicus (rat)** | 0.03891908 | 9 | 134 | 299 | 8790 | 1.409837 | 0.067164 | ***Bdnf//Efna2//Fgf7//Il1b//Insr//Map3k11//Myc//Nfkb2//Pla2g4b*** |
| PPAR signaling pathway - Rattus norvegicus (rat) | 0.03910118 | 4 | 134 | 84 | 8790 | 1.40781 | 0.029851 | *Acsbg1//Cyp27a1//Dbi//Lpl* |
| Serotonergic synapse - Rattus norvegicus (rat) | 0.04323411 | 5 | 134 | 126 | 8790 | 1.364174 | 0.037313 | *Alox12e//Cyp2c22//Htr2b//Kcnj9//Pla2g4b* |
| Gap junction - Rattus norvegicus (rat) | 0.04513993 | 4 | 134 | 88 | 8790 | 1.345439 | 0.029851 | *Htr2b//Prkg2//Tuba1b//Tubb6* |
| **The top ten most significant enrichment pathways and the related details were shown** | | | | | | | | |

Additional file 1: Table S3. GO analysis of increased BRD4-binding peaks in rat TMJ cartilage after overloading mechanical stress.

| GO Term | Count | Fold.Enrichment | P value | Enrichment.Score | Gene.Ratio | Genes |
| --- | --- | --- | --- | --- | --- | --- |
| **regulation of stress-activated MAPK cascade** | 9 | 2.661374 | 0.007127 | 2.147124 | 0.03169 | ***Map3k11//Myc//Il1b//Tlr6//Il1rn//***  ***Sfrp5//Fktn//Sfrp4//Zc3h12a*** |
| chronic inflammatory response | 3 | 6.485187 | 0.010801 | 1.966533 | 0.010563 | *Il1b//Il1rn//Bdnf* |
| leukocyte aggregation | 2 | 10.44836 | 0.015059 | 1.822199 | 0.007042 | *Il1b//Sema4d* |
| regulation of cytokine production involved in inflammatory response | 3 | 5.699104 | 0.015393 | 1.812668 | 0.010563 | *Zc3h12a//Cd6//Tlr6* |
| stress-activated MAPK cascade | 9 | 2.331452 | 0.01594 | 1.797504 | 0.03169 | *Map3k11//Myc//Il1b//Tlr6//Il1rn//*  *Sfrp5//Fktn//Sfrp4//Zc3h12a* |
| cytokine production involved in inflammatory response | 3 | 5.224178 | 0.019454 | 1.71098 | 0.010563 | *Zc3h12a//Cd6//Tlr6* |
| **The top six most significant enrichment GO terms which are related to inflammation and the details were shown.** | | | | | | |

Additional file 1: Table S4. The details of 17 genes with increased BRD4 and H3K27ac overlapped bindings in the promoter region after overloading mechanical force.

| Names | Total | Elements | Details |
| --- | --- | --- | --- |
| Brd4 and H3K27ac  binding sites | 17 | *Csta* | cystatin A  Desmoplakin (DSP) and cystatin A (CSTA) interaction was found in human osteoarthritis.^1^ |
|  |  | *Atp8b1* | ATPase phospholipid transporting 8B1 |
|  |  | *RGD1359290* | Ribosomal_L22 domain containing protein RGD1359290 |
|  |  | *Robo1* | Roundabout guidance receptor 1 |
|  |  | *Cad* | carbamoyl-phosphate synthetase 2, aspartate transcarbamylase, and dihydroorotase  Cad may be regulated by the MAPK pathway ^2^ |
|  |  | *Arfip1* | ADP-ribosylation factor interacting protein 1 |
|  |  | *Zbbx* | zinc finger, B-box domain containing |
|  |  | *Mir181d* | microRNA 181d  *Mir-181d* can inhibit the differentiation of hBMSCs into osteoblasts by regulating the expression of SMAD3.^3^ |
|  |  | *Olr1746* | olfactory receptor 1746 |
|  |  | *Sqor* | sulfide quinone oxidoreductase |
|  |  | *Il1rn* | interleukin 1 receptor antagonist |
|  |  | *Trem1* | triggering receptor expressed on myeloid cells 1  TREM-1 plays a critical in OA development through regulation of NF-κB signaling.^4^  TREM1 was strongly up-regulated in inflamed compared with normal/reactive areas of osteoarthritis synovial membrane.^5^ |
|  |  | *Mcemp1* | mast cell-expressed membrane protein 1 |
|  |  | *Mir181c* | microRNA 181c  Circulating *MiR-181c-5p* and *MiR-497-5p* Are Potential Biomarkers for Prognosis and Diagnosis of Osteoporosis.^6^  NEAT1/*MiR-181c* Regulates Osteopontin (OPN)-Mediated Synoviocyte Proliferation in Osteoarthritis.^7^  *Mir-181a* promotes osteoblastic differentiation through repression of TGF-β signaling molecules.^8^ |
|  |  | *Tmprss11d* | transmembrane serine protease 11D |
|  |  | *Prlhr* | prolactin releasing hormone receptor |
|  |  | *Nanos3* | nanos C2HC-type zinc finger 3 |

Supplemental References

1. Wang, M, Liu, C, Zhang, Y, Hao, Y, Zhang, X, and Zhang, YM (2013). Protein interaction and microRNA network analysis in osteoarthritis meniscal cells. *Genetics and molecular research : GMR* **12**: 738-746.

2. Sigoillot, FD, Evans, DR, and Guy, HI (2002). Growth-dependent regulation of mammalian pyrimidine biosynthesis by the protein kinase A and MAPK signaling cascades. *The Journal of biological chemistry* **277**: 15745-15751.

3. Xie, Y, Hu, JZ, and Shi, ZY (2018). MiR-181d promotes steroid-induced osteonecrosis of the femoral head by targeting SMAD3 to inhibit osteogenic differentiation of hBMSCs. *European review for medical and pharmacological sciences* **22**: 4053-4062.

4. Tang, J, and Dong, Q (2017). Knockdown of TREM-1 suppresses IL-1beta-induced chondrocyte injury via inhibiting the NF-kappaB pathway. *Biochemical and biophysical research communications* **482**: 1240-1245.

5. Lambert, C, Dubuc, JE, Montell, E, Verges, J, Munaut, C, Noel, A*, et al.* (2014). Gene expression pattern of cells from inflamed and normal areas of osteoarthritis synovial membrane. *Arthritis & rheumatology (Hoboken, NJ)* **66**: 960-968.

6. Ma, J, Lin, X, Chen, C, Li, S, Zhang, S, Chen, Z*, et al.* (2020). Circulating miR-181c-5p and miR-497-5p Are Potential Biomarkers for Prognosis and Diagnosis of Osteoporosis. *The Journal of clinical endocrinology and metabolism* **105**.

7. Wang, Q, Wang, W, Zhang, F, Deng, Y, and Long, Z (2017). NEAT1/miR-181c Regulates Osteopontin (OPN)-Mediated Synoviocyte Proliferation in Osteoarthritis. *Journal of cellular biochemistry* **118**: 3775-3784.

8. Bhushan, R, Grunhagen, J, Becker, J, Robinson, PN, Ott, CE, and Knaus, P (2013). miR-181a promotes osteoblastic differentiation through repression of TGF-beta signaling molecules. *The international journal of biochemistry & cell biology* **45**: 696-705.
